# Retention rate of giomer S-PRG filler containing pit and fissure sealant applied with or without etching: a randomized clinical trial

**DOI:** 10.1186/s12903-024-05096-7

**Published:** 2024-11-07

**Authors:** Manar Abdelmageed Elmokanen, Hadier Mahmoud Ahmed Gad

**Affiliations:** Department of Conservative Dentistry, Faculty of Dentistry, Cairo University, Cairo, Egypt

**Keywords:** Pit and fissure sealant, Giomer, Etching, Retention

## Abstract

**Background:**

Pit and fissure sealant is a micro-invasive modality for treating deep and retentive pits and fissures to prevent and/or stop occlusal caries. Every effort needs to be done to enhance sealant retention and survival. The aim of this research is to assess the retention rate of giomer S-PRG filler containing pit and fissure sealant applied with or without etching.

**Methods:**

Overall, 52 patients were included in this trial and they were allocated randomly in 2 groups (*n* = 26). Group 1 (control group) received giomer sealant (Beautisealant, Shofu, Kyoto, Japan) without an etching step, and group 2 (intervention group) received the same but with a separate preparatory etching step before the sealant application. The retention rate of the sealant was assessed over one year at 3 months, 6 months, and 12 months. Intergroup comparison between categorical variables was done using the chi square test, while intragroup comparison was done using Cochran’s q test followed by multiple comparisons. Relative risk was used to evaluate the clinical significance. Survival rate was analyzed using Kaplan-meier and Log-rank test.

**Results:**

Intergroup comparison between both techniques revealed statistically significant difference within different follow up periods (*P* < 0.05). Intragroup comparison within both giomer sealant with etching group and giomer sealant without etching group revealed statistically significant difference between different follow-up periods (*P* < 0.001). There was 69% less risk for total loss or partial loss of giomer sealant with etching when compared to giomer sealant without etching after 12 months.

**Conclusion:**

An initial preparatory step of etching to the enamel surface is crucial before giomer sealant application to enhance its bonding, increase its retention and promote its survival.

**Trial registration:**

Trial registration ClinicalTrials.gov, NCT04929782. Registered 14 July 2024 - Retrospectively registered, https://clinicaltrials.gov/study/NCT06509451

## Background

Dental caries is still a major global health concern that affects both adults and children, despite recent trends showing a decline in its frequency. Demineralization in young permanent teeth fissures frequently occurs after early teeth eruption which increases the risk of dental caries developing. The intricate anatomical architecture of the fissure is the primary cause of dental caries development because it creates the perfect environment for the buildup of food debris and bacterial growth [1].

One of the best non-invasive methods for treating deep, retentive pits and fissures and high caries-risk youngsters is to use pit and fissure sealants to prevent and/or stop occlusal caries [2].

Dental sealants come in a variety of types and brands. Dental sealants come in a variety of types and brands. The most commonly used material for sealing occlusal pits and fissures appears to be either resin based or glass ionomer based sealant [3]. Not only there are several dental materials that can be utilized as sealants, but there are also various methods or techniques of application [4].

The sealant works by bonding the resinous material to an acidetched enamel, which occludes the pits and fissures physically from the surrounding oral environment, but unfortunately over time, the bond between the sealant and the tooth may weaken, which could explain why the fissure sealants fail to remain in place [2].

Even though adhesion has improved, manufacturers have attempted to make the sealant application process simpler and they claimed that it would be simpler to omit the preparatory etching, rinsing and drying steps because these steps can be uncomfortable for patients or challenging for some patients, particularly young ones. To overcome such drawbacks, sealants using self-etch adhesive systems were introduced to simplify such procedures [4]. Self-etching adhesive systems’ primary benefit is their ability to shorten chair times and minimize patient technique sensitivity, two factors that are crucial when treating younger patients [5].

In spite of these advantages of using a self adhesive sealant, such sealants have trouble adhering to previously unprepared or unetched enamel, necessitating proper follow-up and resealing [6].

The primary reason for the retention failure of self-adhesive sealants is that the acidic primer’s lower degree of enamel demineralization compared to phosphoric acid conditioning results in a lesser degree of aprismatic enamel removal, which lowers micromechanical imbrication and affects sealant adhesion [7].

One of the introduced sealants with self-etch primer is the giomer sealant that includes S-PRG fillers that releases multiple ions like fluoride, sodium, aluminum, strontium, borate and silicate. The ions released can buffer acids, prevent demineralization, encourage remineralization, and prevent plaque formation [8]. Fluoride and strontium react with hydroxyapatite to generate fluoro-apatite and strontium-apatite, strengthening the structure of the tooth and forming an acid-resistant coating. It is also known that S-PRG fillers can continuously release and absorb fluoride [9]. Although the manufacturer’s instructions for placement of giomer sealant don’t recommend a separate etching step, it was found that the giomer sealant retention that used a self-etching primer was poor and this could be due to the absence of the cleaning etching step [10].

To date, there is no trial in the literature that investigated the effect of etching on the retention rate of giomer S-PRG filler containing pit and fissure sealant. Accordingly, this trial tried to modify the clinical application technique of the intervention group by receiving the giomer sealant after a preparatory etching step to enhance the retention to the tooth enamel surface.

The aim of this trial is to assess the retention rate of giomer sealant applied with or without etching over one year. The null hypothesis is that giomer sealant applied either with or without etching will have the same retention rate.

## Methods

### Study settings

This study was conducted at the Conservative Dentistry Department, Faculty of Dentistry, Cairo University in Egypt. All procedures done in this trial were in agreement with the ethical standards of the Research Ethics Committee of the Faculty of Dentistry, Cairo University (CREC) with ethical approval number (37124). Signed informed consent was signed by each participant. The protocol of this trial was registered in (www.clinicaltrials.gov), with identification number (NCT06509451).

### Sample size calculation

The sample size was calculated depending on a previous trial by Penha et al. in 2021 in which total loss within bioactive self-etch sealant group happened in 75% of cases. By implementing a two tailed Z test for difference between two independent proportions with an alpha level of 5% and a power of 80%. The minimum sample size needed was 24 per group in order to detect a difference of 40%. Sample size was increased by 10% [11 & 12] to compensate for possible dropouts to reach 26 teeth per group. Sample size was performed using G*Power version 3.1.9.2 for windows.

### Materials

Materials used in this trial (Table 1) were the Giomer S-PRG filler containing pit and fissure sealant (Beautisealant, Shofu, Kyoto, Japan), its self etching primer (Beauisealant primer, Shofu, Kyoto, Japan) and phosphoric acid etchant gel (Vococid, Voco, GmbH, Germany).

**Table 1:** Materials’ specification, composition and manufacturer

Material	Specification	Composition	Manufacturer
Beautisealant	Giomer S-PRG filler containing sealant	S-PRG filler (30% by weight), microfurned silica, UDMA	Shofu, Kyoto, Japan
Beautisealant Primer	Self etching primer	Acetone, Phosphoric acid monomer, carboxylic acid monomer, distlled water	Shofu, Kyoto, Japan
Vococid	Etching gel	35% phosphoric acid with silica thickener and blue dye for visual control	Voco, GmbH, Germany

### Recruitment, study design and grouping

This clinical trial was a randomized, controlled, prospective, double-blinded study with two parallel arms and allocation ratio 1:1. The study was carried out in the Faculty of dentistry, Cairo University. They were recruited from the patients’ flow at the Conservative Dentistry Department clinics at Faculty of Dentistry, Cairo university, where there is a high and continuous flow of eligible patients. All personal, medical, and dental histories were taken from each patient and documented in their diagnostic charts. All participants signed an informed consent prior to the initiation of the trial containing all the ethical aspects of the trial. The 52 patients assigned to this trial were allocated randomly to 2 groups (*n* = 26), where group 1 (control) received giomer sealant without an etching step, and group 2 (intervention group) received the same giomer sealant but with a separate etching step before the sealant application.

### Eligibility criteria

Patients recruited in this trial were fulfilling certain eligibility criteria. Both the inclusion and exclusion criteria are shown in (Table 2).

**Table 2:** Inclusion and exclusion criteria of the participants

Inclusion criteria	Exclusion criteria
1. ICDAS score 0 or 1 2. Satisfactory oral hygiene 3. Normal saliva (consistency, pH, flow rate, buffering capacity) 4. Cooperative attitude 5. Age between 15–30 years old 6. Males or females 7. Participants signed the informed consent	1. ICDAS score > 1 2. Poor oral hygiene 3. Evidence of tempromandibular joint disorders or malocclusion

### Randomization and sequence generation

Sequence generation was created by simple randomization through arranging numbers from 1:52 using Random Sequence Generator, Randomness, and Integrity Services Ltd (https://www.random.org/). Each created number randomly represented a group in a random manner, that is, No. 1 control group (from 1 to 26) and No. 2 intervention group (from 27 to 52). Then, the patients are asked to choose between numbers in opaque sealed envelopes, which contained the randomization code.

### Blinding

The study conducted is a double-blinded study. The assessors and the participants were blinded to the technique of the material applied. However, the operator can’t be blinded because there was difference between the control and intervention groups in the technique of the material application, with an extra step of etchant application in the intervention group.

### Control group

Regarding the control group, a thorough prophylaxis of the teeth was first performed, using a rotatory brush and pumice, a step that reduces microleakage and improves the sealant retention [13]. Then, Beautisealant primer adequate amount was applied to enamel using a microbrush, kept for five seconds undisturbed and then gently dried with air to avoid its removal. Beautisealant was directly applied from the syringe inside the occlusal fissures through disposable tips and light-cured for ten seconds, according to the manufacturer’s instructions [4].

### Intervention group

Regarding the intervention group, the same steps were done except for an additional initial etching step with orthophosphoric acid gel 35% (Vococid) for thirty seconds (for uncut enamel). Teeth were washed using air-water spray for thirty seconds, then dried with air free from oil for fifteen seconds, then checked for appropriate etching before primer and sealant application [14].

### Outcome assessment

Two calibrated and blinded assessors assessed the applied pit and fissure sealant. Before the study initiation, in vitro and in vivo standardization was done to calibrate both assessors. In vitro calibration was done through theoretical discussion about Simonsen scoring system that would be used, in addition to photographic analysis. In vivo calibration was done by examining 25 patients with previously applied sealants who had not participated in the study, then re-examining same patients and comparing the observations after a 14 days interval. Intra examiner reliability was tested with intra-class correlation coefficient measuring 1 and inter examiner reliability also was tested with kappa co-efficient measuring 0.84, so both assessors were competent to conduct the study. The assessment was blind, as sealant application technique of each patient was not informed to both assessors. All sealants were assessed at 3, 6 and 12 months.

The clinical evaluation criteria were related to the retention of the material, according to Simonsen criteria [15]. Tooth occlusal surface was evaluated by using a dental mirror and explorer and sealant retention was scored according to the following criteria:

1. Total retention (TR): all pits and fissures of the occlusal surface are covered and no material ledge.
2. Partial loss (PL): fracture and/or some loss of material.
3. Total loss (TL): absence of material from the occlusal surface.

### Statistical analysis

Data was analyzed using Medcalc software, version 22 for windows (MedCalc Software Ltd, Ostend, Belgium). Categorical data was shown as frequency and percentage. Intergroup comparison between categorical variables was done using the chi square test, while intragroup comparison was done using Cochran’s q test followed by multiple comparisons. Relative risk was used to evaluate the clinical significance. Survival rate was analyzed using Kaplan-meier and Log-rank test. A P value less than or equal to 0.05 was regarded statistically significant for intergroup comparison, while for intragroup comparison P value was Bonferroni corrected (*P* ≤ 0.0083) and all tests were two tailed.

## Results

### 1. Demographic data

**Fig. 1:**
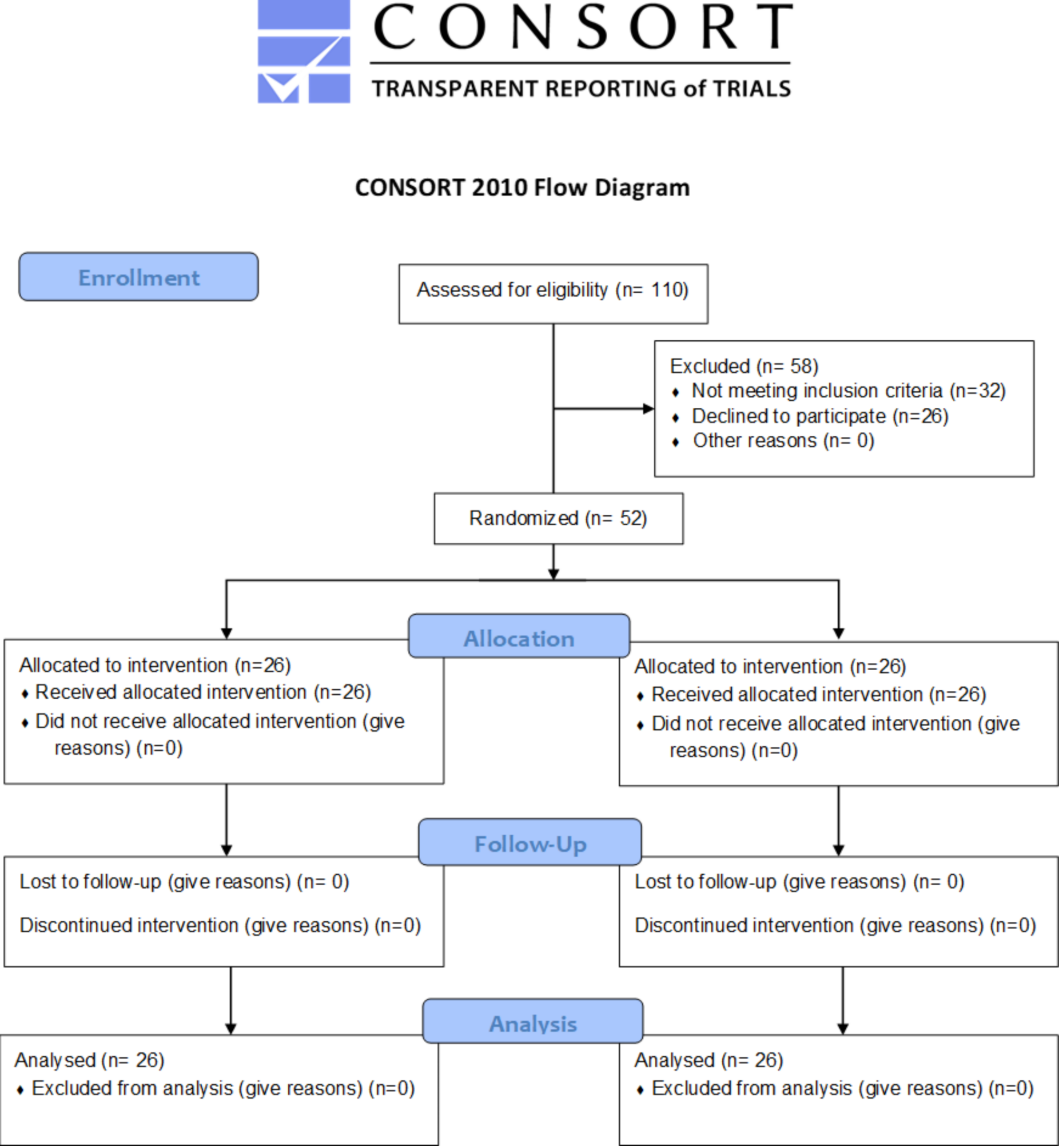
Consort flow diagram

The current study was carried out on (52) teeth that were allocated randomly to the control and intervention groups. After 12 months, 52 teeth were evaluated with 100% retention rate. The total number of participants assessed for eligibility, recruitment, randomization, allocation, and evaluation is represented in the CONSORT flow diagram (Fig. 1).

Gender distribution is shown in (Table 3), there was no statistically significant difference between both groups regarding gender (*P* = 0.4011). Mean age of the patients in this trial was 17.5 ± 1.9 years; mean age in the intervention group was 17.8 ± 1.9 years, while in the control group mean age was 17.2 ± 1.8 years, there was no statistically significant difference between both groups regarding age (*P* = 0.193). Teeth distribution is shown in (Table 4), there was no statistically significant difference regarding teeth distribution between both groups (*P* = 0.3270).

**Table 3:** Gender distribution among groups

Sealant	Gender		Row Total (RT)
	Males	Females	
Giomer with etching	9 34.6% RT 42.9% CT	17 65.4% RT 54.8% CT	26 (50.0%)
Giomer without etching	12 46.2% RT 57.1% CT	14 53.8% RT 45.2% CT	26 (50.0%)
Column total (CT)	21 (40.4%)	31 (59.6%)	52
P value	*P* = 0.4011		

**Table 4:** Teeth distribution among groups

Sealant	Distribution				Row Total (RT)
	Maxillary 1st molars	Maxillary 2nd molars	Mandibular 1st molars	Mandibular 2nd molars	
Giomer with etching	9 34.6% RT 47.4% CT	4 15.4% RT 40.0% CT	10 38.5% RT 50.0% CT	3 11.5% RT 100.0% CT	26 (50.0%)
Giomer without etching	10 38.5% RT 52.6% CT	6 23.1% RT 60.0% CT	10 38.5% RT 50.0% CT	0 0.0% RT 0.0% CT	26 (50.0%)
Column total (CT)	19 (36.5%)	10 (19.2%)	20 (38.5%)	3 (5.8%)	52
P value	*P* = 0.3270				

### 2. Retention

Intergroup comparison between both techniques revealed statistically significant difference along different follow up periods; baseline, 3, 6 and 12 months (*P* < 0.05). Intragroup comparison in giomer with etching group or giomer without etching group has revealed statistically significant difference between different follow-up periods (*P* < 0.001). There was 69% less risk for total loss or partial loss of giomer with etching when compared to giomer without etching after one year (RR = 0.3158 (95% CI 0.1507 to 0.6616; *P* = 0.0023)). After 12 months giomer with etching have shown total retention in 61.5% of sealants, while giomer without etching showed total retention in only 26.9% of sealants.

Total retention was evident more in mandibular molars after 6 and 12 months (63% and 78%) respectively when compared to maxillary molars (37% and 22%) respectively, showing a statistically significant effect of tooth position on retention rate (*P* < 0.05). On the contrary, gender had no statistically significant effect on retention rate (*P* > 0.05) (Table 5).

**Table 5:** Frequency and percentage of retention for intergroup and intragroup comparison between materials within each follow-up

Follow-up	Giomer sealant with etching				Giomer sealant without etching				*P* value
	TR	PL	TL	Rank	TR	PL	TL	Rank	
Baseline	26(100%)	0(0%)	0(0%)	A	26(100%)	0(0%)	0(0%)	A	*P* = 1.0000*
3 months	25(92.2%)	0(0%)	1(3.8%)	A	13(50%)	3(11.5%)	10(38.5%)	B	*P* = 0.0008*
6 months	20(76.9%)	2(7.7%)	4(15.4%)	AB	10(38.5%)	4(15.4%)	12(46.2%)	B	*P* = 0.0183*
12 months	16(61.5%)	4(15.4%)	6(23.1%)	B	7(26.9%)	4(15.4%)	15(57.7%)	B	*P* = 0.0250*
P value	*P* < 0.001*				*P* < 0.001*				

### 3. Survival analysis

**Fig. 2:**
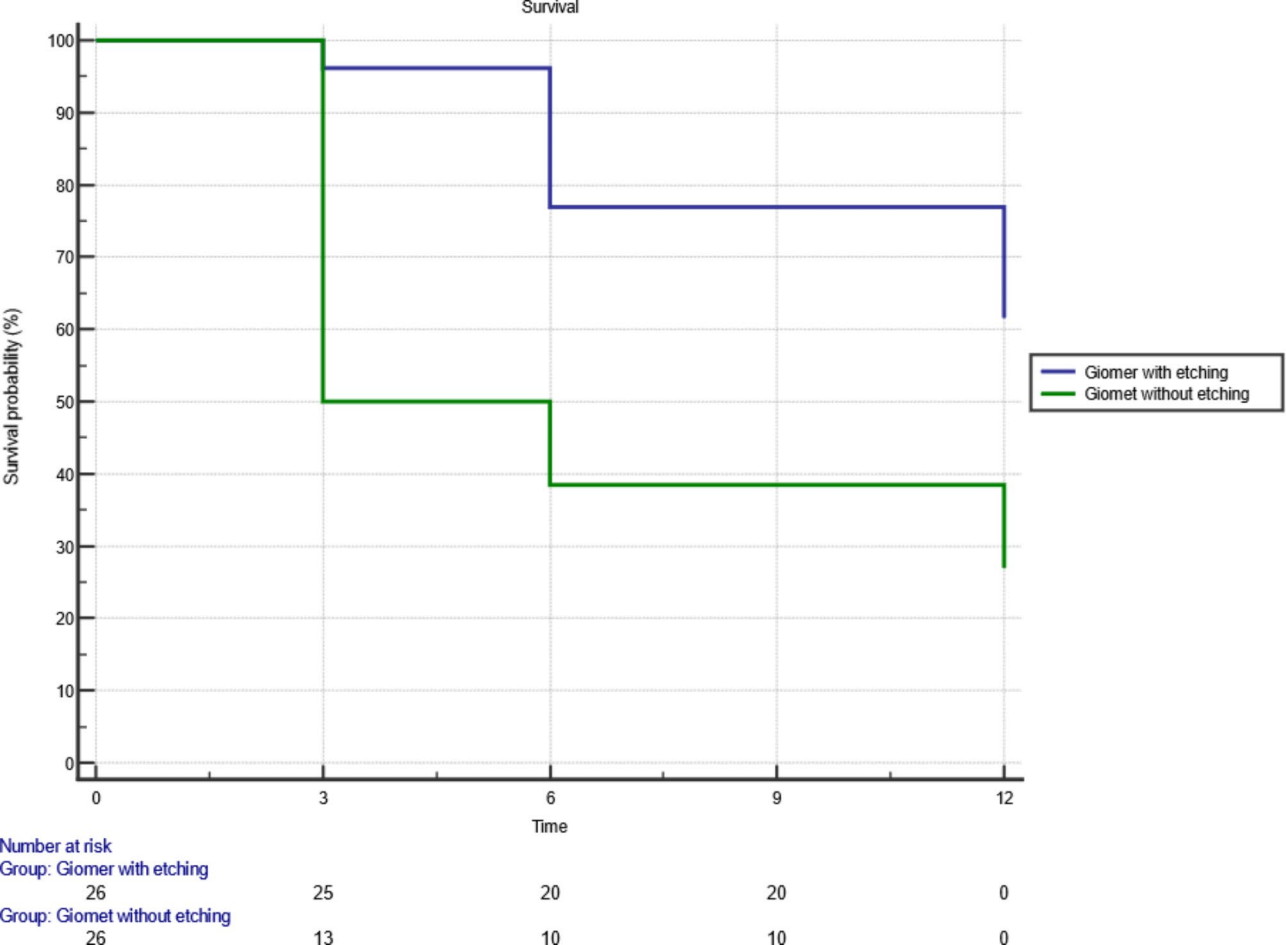
Survival analysis of both sealants after 12 months

Overall sealants’ survival was evaluated after 12 months, 10 sealants in giomer with etching group and 19 sealants in giomer without etching group failed after one year either due to partial or total loss. To obtain survival curves, Kaplan-meier analysis was used. To compare between survival curves, Logrank test was used. There was statistically significant difference between both sealants survival (*P* = 0.0026). (Fig. 2)

## Discussion

In this trial the retention rate was the assessed outcome because the sealant retention is commonly regarded as an indicator for caries prevention, so it is considered as the main and most crucial outcome for the assessment of the success of pit and fissure sealant [16].

Giomers are hybrid materials obtained by combining the characteristics of the resin composite and glass-ionomers [17]. Giomer pit and fissure sealant contains S-PRG fillers. The S-PRG manufacturing process is based on a technology in which grinding and heat treatment of fluoroaluminosilicate glass ionomer powder. Then, it is mixed with polyacrylic acid to form a glass ionomer phase stable on glass surface [18]. Due to having this glass ionomer phase on the surface, giomer materials have both the beneficial characteristics of glass ionomer and the strength of conventional resin composite material. This S-PRG containing material is biofunctional, because it releases multiple ions, in addition to the sealing ability [1].

The ion release, remineralizing and antibacterial effect of the giomer sealant could be so beneficial for a sealant to do its function [19]. However, being a self-etch sealant with no preparatory step for the tooth enamel interferes with its retention and accordingly its survival rate. As it was reported by the groundbreaking trials done by Buonocore that since pit and fissure sealants were introduced, their application was based, on acid etching which enhance the bonding of resin materials to tooth enamel [2]. Accordingly and typically, a preliminary phosphoric acid etching step of enamel before sealant application is mandatory to clean the tooth surface and improve its bonding [1].

When adhesive systems are applied over etched enamel during the sealant placement process, the etched enamel’s inter and intra-prismatic boundaries can be optimally infiltrated and encapsulated, and lengthy adhesive tags can formed. This creates a micromechanical protective barrier on the fissure surface [5]. It was found that the retention rate is better in conventional resin sealants. However, the caries control was found to be better in the giomer sealant [1]. Accordingly, this trial tried to enhance the retention rate of the giomer sealant, by doing a separate etching step, in order to combine both the high clinical performance and survival rate with the high caries control of the S-PRG filler containing sealant.

The results of the current trial showed that there was statistically significant difference between both techniques regarding the retention rate within different follow up periods and that there was 69% less risk for total loss or partial loss of giomer sealant with etching when compared to giomer sealant without etching after 12 months, which means that the etching step enhanced the sealant retention and promoted its survival. Accordingly, the null hypothesis of this study is rejected.

This finding is in agreement with **Singh et al.**,** 2022** who assessed the retention rate of giomer sealant applied with its self-etch primer and found about 74% total loss after one year follow up, which means that only one fourth of the sample size has total sealant retention [20].

Also, **Penha et al.**,** 2021** assessed the retention of the Beautisealant and found that it has poor retention over 12 months period with low survival rate and the high loss risk. They clarified that this may be due to the poor enamel demineralization of the weak primer than the acid etchant that can remove aprismatic enamel. They were in favor of the idea of enamel etching as it enhances not only wetting, adhesion and retention rates of the sealant, but also the bioactive effects which should be investigated in future clinical trials [7].

Moreover, **Özgür et al.**,** 2022** found that after one year, the retention rate of Beautisealant was only %8. This high failure rate found in this giomer sealant was clarified by the probable limitation in the etching ability of the self-etch primer [14].

In addition, **Althomali et al.**,** 2022** assessed the retention of fissure sealant applied by self-etch versus conventional acid-etch techniques and found that within 2 years, the retention of the conventional acid-etching technique was better than the self-etch technique. The great loss of sealants treated with self-etch technique was explained by the low pH of self-etch adhesives that cannot efficiently etch the uncut enamel and accordingly makes incomplete resin penetration into enamel to create resin tags that weakens the bond strength of the sealant. The etching step is primarily done for smear layer removal, selective enamel rods’ dissolving, and macro/microporosities formation. Hydrophobic resin material penetration occurs by capillary attraction and upon light curing, the micromechanical interlocking occurs [10].

**Amend et al.**,** 2021** also mentioned that after phosphoric acid etching introduction by Buonocore in 1955, enamel etching is considered the gold standard to achieve strong bond by resin micromechanical retention. The phosphoric acid application exposes prismatic enamel, creates microporosities that allows the penetration of the resin-based sealants and upon polymerization, mechanical interlocking occurs through resin tags formation [21].

This trial results support the conclusion of the systematic review and meta-analysis done by **Botton et al.**,** 2016** who found that fissure sealants applied using self-etch approach show lower retention than sealants applied using the conventional one [3].

However, the finding of this trial was contradicted by **Ogawa et al.**,** 2022** who found that the application of Beautisealant does not need etchant pretreatment to enhance the sealant retention, explaining that although the application of an etchant cleans the enamel and enhances adhesion, but simultaneously it leads to excessive enamel demineralization. They added that Beautisealant is effective in reducing enamel demineralization through S-PRG fillers that enhance remineralization due to ions in their composition, like fluoride [1].

In addition, **de Souza Penha et al.**,** 2023** found that over one year follow-up period, there was no significant difference in the retention of the giomer sealant that use the self-etch technique versus a conventional resin sealant that use the etch and rinse technique [15].

Regarding the teeth treated, the results of this trial revealed that total sealant retention was evident more in mandibular molars after 6 and 12 months (63% and 78%) respectively when compared to maxillary molars (37% and 22%) respectively, showing a statistically significant effect of tooth position on retention rate (*P* < 0.05).

This is in agreement with **Reddy et al.**,** 2015** who found that sealant retention on the maxillary teeth was less than mandibular teeth. They explained that the enhanced sealant retention in lower teeth may be due to direct vision and easier sealant flow aided by the gravity. Also, the action of occlusal stress on the sealant of the upper molar occurs at an earlier stage of eruption than that of the lower molar. In the early stages of mandibular eruption, the upper teeth touch only lower molars’ cusps without touching the sealant [22].

This is also in accordance with **Ratnaditya**,** et al. 2015**, whose study found higher sealant retention rates for lower teeth which could be due to the direct visualization that makes the sealant application easier and improved without much visual errors. In addition, easy flow of the sealant, aided by the gravity and the well-defined pits and fissures [13].

However, this finding is disagreed by **Muntean et al. 2021**, who found in their study that maxillary molars showed better retention than mandibular molars after one year and added that the repair rate is more in lower teeth, because their eruption periods last for longer time. But this finding may be due to the younger age group of their participants, with 7 years old average age. Accordingly, the poor sealant retention in the young children mandibular molars that needed more retreatment is due to having a distal tissue flap or operculum that lasts for a long period, so making the occlusal surface isolation of mandibular molars more difficult and causing the sealant poor retention subsequently [16].

The limitation of this trial is lack of prior research studies on this topic and the need for a longer term clinical follow-up to ensure the effect of etching on giomer pit and fissure sealant retention enhancement comparing it with resin based sealants.

To sum up, although using a self adhesive sealant can reduce the chair time and patient technique sensitivity [5], this will be of no benefit if the sealant has poor retention and unfortunately is lost either partially or totally. On the other side, adding an initial preparatory etching step for the enamel surface as done in this trial, despite increasing the application time for few minutes, this enhances the bonding and retention of the giomer sealant and promotes its survival as the results of this trial revealed.

Accordingly, when comparing the pros and cons of each technique, it is preferred to go for an enamel etching step before the application of giomer S-PRG filler containing pit and fissure sealant, although it is not recommended by the manufacturer.

## Conclusion

An initial preparatory step of etching to the enamel surface is crucial before the application of the giomer S-PRG filler containing pit and fissure sealant to enhance its bonding, increase its retention and promote its survival.

### Recommendation

To our knowledge, this is the first trial to assess the effect of etching on the retention rate of giomer pit and fissure sealant, so more clinical trials are needed to ensure the finding of this trial. Moreover, more trials are needed to assess other methods or techniques that would enhance the retention rate of giomer pit and fissure sealant like fissurotomy or air abrasion.

## Data Availability

Data is provided within the manuscript or supplementary information files and it is available from the corresponding author manar_ahmed@dentistry.cu.edu.eg on reasonable request.

## Abbreviations

S- PRG: surface pre-reacted glass-ionomer
ICDAS: international caries detection and assessment system
TR: total retention of sealant
PL: partial loss of sealant
TL: total loss of sealant
